# Irreversible Electroporation Versus Radiofrequency Ablation for Hepatic Malignancies: A Systematic Review and Meta-Analysis

**DOI:** 10.7759/cureus.111579

**Published:** 2026-06-26

**Authors:** Mehreen Iqbal, Sathish Narayanaswamy, Muhammad Shoaib Anwar, Uday Shree Akkala Shetty, Anastasia Postoev, Sandipkumar S Chaudhari, Rahman Hameed Mohammed Abdul, Danish Allahwala

**Affiliations:** 1 Ophthalmology, Rochdale Infirmary, Rochdale, GBR; 2 Neurosciences, University Hospitals of North Midlands, Stoke-on-Trent, GBR; 3 Pharmacology and Therapeutics, Combined Military Hospital Lahore and Institute of Dentistry, Lahore, PAK; 4 Internal Medicine, Southern Regional Medical Center, Riverdale, USA; 5 Internal Medicine, Caribbean Medical University, Willemstad, CUW; 6 Cardiothoracic Surgery, University of Alabama at Birmingham, Birmingham, USA; 7 Family Medicine, University of North Dakota School of Medicine and Health Sciences, Fargo, USA; 8 Gastroenterology and Hepatology, King's Mill Hospital, Sutton-in-Ashfield, GBR; 9 Nephrology, Fatima Memorial Hospital, Karachi, PAK

**Keywords:** hepatic malignancies, irreversible electroporation, radiofrequency ablation, recurrence, systematic review and meta-analysis

## Abstract

The aim of this study was to systematically pool all direct comparative evidence from two-arm studies comparing irreversible electroporation (IRE) and radiofrequency ablation (RFA) for hepatic malignancies and evaluating local tumour recurrence/progression (LTRP), complication rates, and overall mortality. PubMed/MEDLINE, EMBASE, Web of Science, and the Cochrane Library were systematically searched from inception to April 30, 2026. Only studies with a direct IRE versus RFA comparison arm were eligible. Six direct comparative studies were included (two randomised controlled trials (RCTs) and four retrospective cohorts; n=623: IRE=258, RFA=365). Pooled analysis showed no significant difference between IRE and RFA in LTRP (RR 0.68, 95%CI 0.32-1.44; I2=64%; four studies), complication rates (RR 0.98, 95%CI 0.69-1.40; I2=0%; four studies), or mortality (RR 1.02, 95%CI 0.43-2.41; six studies). A consistent non-significant trend towards reduced LTRP with IRE was observed across studies. IRE demonstrates equivalent safety and a consistent, though statistically non-significant, trend towards superior local tumour control compared with RFA for hepatic malignancies. The high heterogeneity in LTRP and the limited number of studies preclude definitive conclusions. Larger, adequately powered RCTs with standardised endpoints are needed to definitively establish the comparative efficacy of IRE in this setting.

## Introduction and background

Primary and secondary hepatic malignancies represent a major global health burden. Hepatocellular carcinoma (HCC) is the sixth most commonly diagnosed cancer worldwide and the third leading cause of cancer-related mortality, with approximately 906,000 new cases and 830,000 deaths annually [1]. Colorectal liver metastases (CRLM) develop in approximately 50% of patients with colorectal cancer, with the liver being the most frequent site of distant metastasis [2]. Together, these account for the majority of the interventional oncology workload globally.

Surgical resection remains the gold standard for both HCC and resectable CRLM; however, only 20-30% of patients are candidates at presentation due to tumour distribution across multiple liver segments, proximity to major vascular or biliary structures, insufficient future liver remnant, underlying liver dysfunction, or comorbidity [3]. Image-guided percutaneous ablation has therefore emerged as an established curative-intent alternative, endorsed by the European Association for the Study of the Liver (EASL), the American Association for the Study of Liver Diseases (AASLD), and the European Society for Medical Oncology (ESMO) guidelines, particularly for tumours ≤3 cm [4].

Radiofrequency ablation (RFA) has been the most widely adopted thermal ablation technique for over two decades, with the largest body of prospective evidence supporting its safety and efficacy [5]. Microwave ablation (MWA) has subsequently gained acceptance, offering larger and more uniform ablation zones and reduced procedural time [6]. Nevertheless, both thermal modalities share a fundamental limitation: efficacy is significantly impaired for tumours adjacent to major hepatic vessels due to the heat-sink effect, in which flowing blood dissipates thermal energy and creates incomplete ablation margins [7]. Peribiliary and subcapsular locations carry additional risk of thermal injury to adjacent structures.

Irreversible electroporation (IRE) was approved by the US Food and Drug Administration in 2009 for soft tissue ablation [8] and overcomes these limitations through a fundamentally different mechanism. IRE delivers a series of short, high-voltage electrical pulses via multiple percutaneous electrodes, creating irreversible nanopores in cell membranes and inducing apoptotic and necrotic cell death without generating sustained thermal injury [9]. Critically, the extracellular scaffolding, bile ducts, and vascular walls are largely preserved, making IRE particularly suited to tumours adjacent to critical hepatic structures [10].

Despite growing adoption, the comparative evidence base between IRE and RFA remains limited. Previous systematic reviews and meta-analyses have predominantly pooled single-arm IRE cohorts without a thermal comparator [11-13], precluding any comparative efficacy or safety inference. The few head-to-head comparative studies available have not been formally synthesised in a meta-analysis restricted to direct two-arm designs.

The aim of this systematic review and meta-analysis is to synthesise all available direct comparative evidence from studies with both an IRE arm and an RFA arm to determine whether IRE is equivalent, inferior, or superior to RFA.

## Review

Methods

Reporting

This systematic review was conducted in accordance with the Preferred Reporting Items for Systematic Reviews and Meta-Analyses (PRISMA) 2020 statement [14]. The protocol of this study was registered with PROSPERO.

Eligibility Criteria

Studies were eligible if they: (1) directly compared IRE (any device) with RFA as the primary ablative modality in a two-arm design; (2) enrolled adult patients with primary or secondary hepatic malignancy; (3) reported at least one pre-specified efficacy or safety outcome; and (4) provided extractable group-level data. Eligible designs included randomised controlled trials (RCTs) and observational cohort studies (prospective or retrospective). Single-arm IRE studies, studies without a direct RFA comparator group, case reports, conference abstracts without full-text data, and preclinical or in vitro studies were excluded. There was no restriction on language or publication date.

Search Strategy

Systematic electronic searches were performed in PubMed/MEDLINE, EMBASE, Web of Science, and the Cochrane Central Register of Controlled Trials (CENTRAL) from database inception to April 30, 2026, using the following terms in combination: ("irreversible electroporation" OR "nanoknife" OR "IRE ablation") AND ("radiofrequency ablation" OR "thermal ablation") AND ("liver" OR "hepatic" OR "hepatocellular carcinoma" OR "liver metastases"). Reference lists of eligible studies and prior reviews were hand-searched. ClinicalTrials.gov and the WHO ICTRP were searched for registered but unpublished trials. Search was conducted by two reviewers independently. Any disagreement between two authors was resolved through discussion.

Study Selection and Data Extraction

Two reviewers independently screened titles and abstracts, followed by full-text assessment of potentially eligible records. Disagreements were resolved by consensus or arbitration by a third reviewer. Data were extracted onto a standardised form capturing study design, country, patient demographics, follow-up, and all reported outcomes. Where outcomes were reported at multiple timepoints, the longest available follow-up was preferred for pooling.

Risk of Bias Assessment

RCTs were assessed using the Cochrane Risk of Bias 2.0 (RoB 2) tool [15] across five domains: randomisation process, deviations from intended interventions, missing outcome data, outcome measurement, and selection of reported results. Cohort studies were appraised using the Newcastle-Ottawa Scale (NOS) [16] with scores of 7-9 considered low risk, 5-6 moderate, and <5 high risk. Two reviewers independently performed all assessments.

Statistical Analysis

Analysis was performed using RevMan 5.4.1. For all outcomes, the risk ratio (RR) was reported with 95% confidence interval (CI). Statistical significance was defined as two-tailed p<0.05. Statistical heterogeneity was assessed using the Cochran Q test (significance threshold p<0.10) and the I-square statistic (0-25% low; 25-50% moderate; 50-75% substantial; >75% considerable). The random-effects model was applied irrespective of the heterogeneity. Given the current evidence base (k=3 to 4), formal publication bias testing was not conducted.

Results

**Figure 1:**
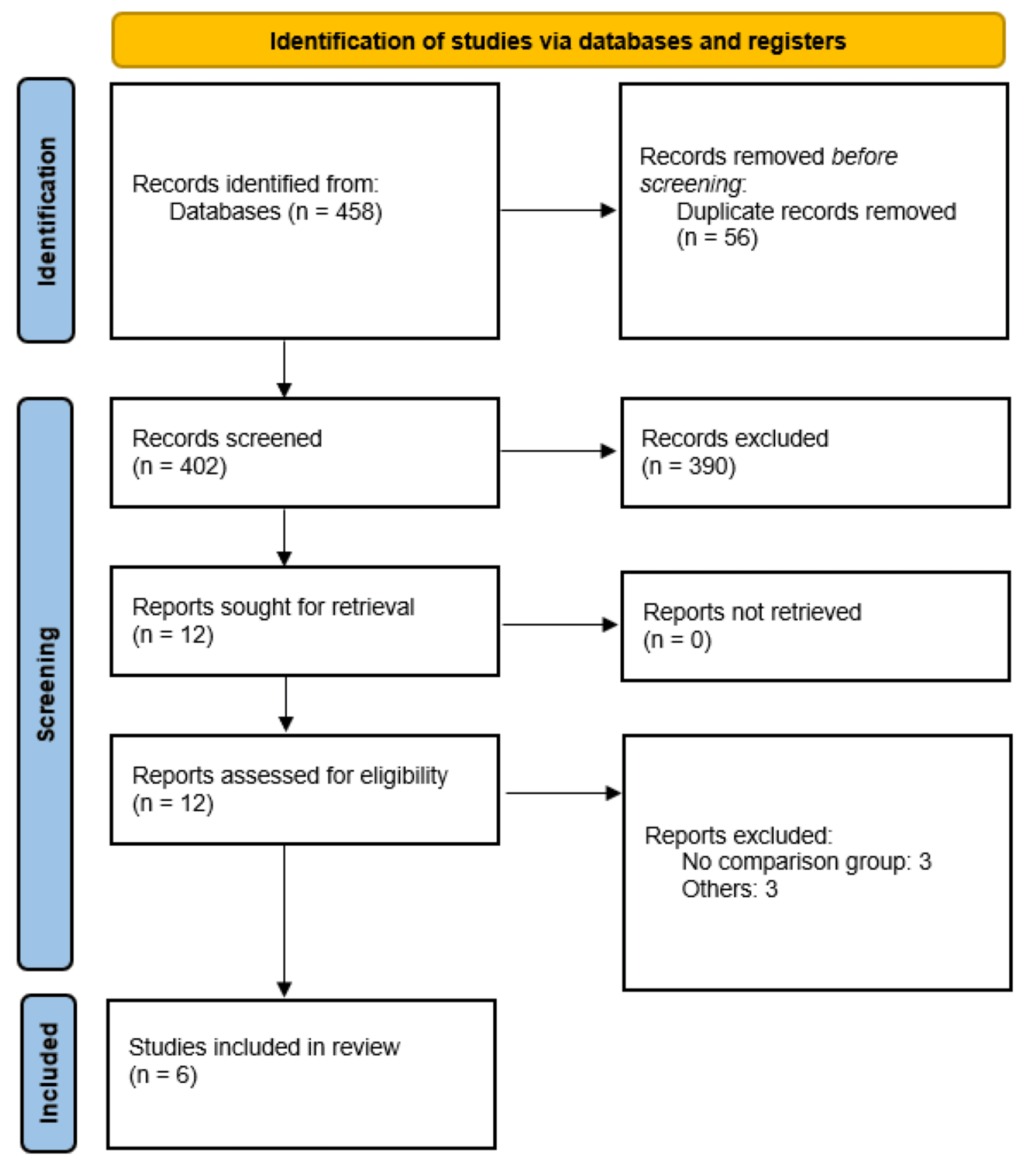
Study selection flowchart (PRISMA) PRISMA: Preferred Reporting Items for Systematic Reviews and Meta-Analyses

The initial database search identified 458 records. After removing duplicates (n=56), 402 unique records were screened at the title and abstract level. Twelve full-text articles were assessed for eligibility, of which 6 were excluded. Six studies met all eligibility criteria [17-22] and are included in this meta-analysis. A PRISMA flow diagram is provided as Figure 1.

The six included studies comprised two RCTs [18,22] and four retrospective cohort studies [17,19-21] published between 2019 and 2025. Combined, 623 patients were included (IRE: 258; RFA: 365). Tumour pathologies included HCC in four studies [17,19-21] and mixed hepatic malignancies (HCC plus metastases) in two [18-22]. Mean tumour sizes ranged from 1.7 to 2.3 cm. Follow-up ranged from six months to 24 months across studies. Full characteristics are presented in Table 1. Tables 2, 3 present the quality assessment of the included studies.

**Table 1:** Characteristics of the included studies HCC: Hepatocellular Carcinoma; RCT: Randomised Controlled Trial; Cohort: Observational Cohort Study; IRE: Irreversible Electroporation; RFA: Radiofrequency Ablation; n: Number of Patients; NR: Not Reported; BCLC: Barcelona Clinic Liver Cancer (Staging System)

Study	Year	Design	Population	IRE n	RFA n	Follow-up
Freeman et al. [17]	2021	Cohort	HCC (single centre)	22	22	NR
Liu et al. [18]	2022	RCT	Malignant hepatic tumours (mixed)	16	16	12 months
Verloh et al. [19]	2019	Cohort	HCC in perivascular/peribiliary locations	47	117	NR
Wada et al. [20]	2023	Cohort	Early-stage HCC (BCLC 0/A)	15	15	NR
Wang et al. [21]	2025	Cohort	Subcapsular HCC (Milan criteria)	80	133	22 months (median)
Zhang et al. [22]	2022	RCT	Solid liver tumours (HCC + metastases, ≤4 cm)	78	74	6 months

**Table 2:** Risk of bias assessment for RCTs RCT: Randomised Controlled Trial

Study	D1 Randomisation process	D2 Deviations from intended	D3 Missing outcome data	D4 Measurement of outcome	D5 Selective reporting	Overall judgement
Liu et al. (2022) [18]	Some concerns	Low	Some concerns	Low	Some concerns	Some concerns
Zhang et al. (2022) [22]	Low	Low	Low	Low	Low	Low risk

**Table 3:** Risk of bias assessment for RCTs RCT: Randomised Controlled Trial

Study	Selection	Comparability	Outcome	Overall
Freeman et al. (2021) [17]	4	2	1	Moderate
Verloh et al. (2019) [19]	4	1	1	Moderate
Wada et al. (2023) [20]	4	2	1	Good
Wang et al. (2025) [21]	4	3	2	Good

Meta-Analysis of Outcomes

**Figure 2:**
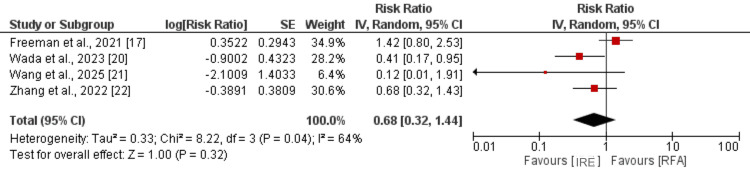
Comparison of local tumour recurrence/progression (LTRP) between the two groups [17,20-22] IRE: Irreversible Electroporation; RFA: Radiofrequency Ablation

Local tumour recurrence/progression (LTRP): Four studies assessed this outcome, and the results are presented in Figure 2. Pooled analysis showed that the risk of developing LTRP was lower in the IRE group compared to its counterpart, but the difference was not statistically significant (RR: 0.68, 95% CI: 0.32 to 1.44). High heterogeneity was reported among the study results (I-Square: 64%).

**Figure 3:**
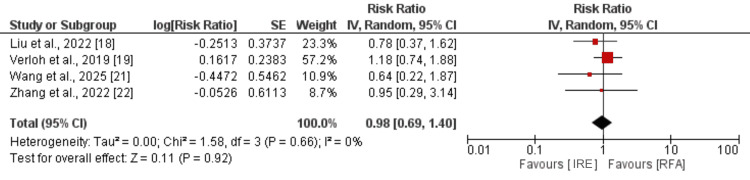
Comparison of complications between the two groups [18,19,21,22] IRE: Irreversible Electroporation; RFA: Radiofrequency Ablation

Complication rate: Four studies compared the complication rate between the two groups. As shown in Figure 3, no significant difference was found between two groups in terms of complication rate (RR: 0.98, 95% CI: 0.69 to 1.40). No heterogeneity was reported among the study results (I-Square: 0%).

**Figure 4:**
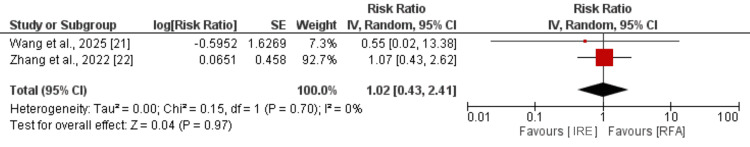
Comparison of mortality between the two groups [21,22] IRE: Irreversible Electroporation; RFA: Radiofrequency Ablation

Mortality: Two studies compared the mortality between the two groups, and the results are shown in Figure 4. Pooled analysis showed that no significant differences in mortality were observed between the two groups (RR: 1.02, 95% CI: 0.43 to 2.41).

Discussion

This systematic review and meta-analysis, which is the first to restrict inclusion exclusively to studies with a direct two-arm IRE versus RFA comparison in hepatic malignancies, identifies three principal findings. First, IRE achieves equivalent overall complication rates to thermal ablation, with highly consistent safety equivalence across heterogeneous study settings. Second, recurrence was lower in IRE compared to RFA, but the differences were statistically insignificant. Third, mortality appears comparable between the two groups. However, considering the fact that a lower number of studies were included in this meta-analysis, findings need to be interpreted with caution. As this is the first meta-analysis comparing two treatment modalities, future research should be conducted to confirm the findings.

Bulvik et al. demonstrated preserved blood flow within vessels following IRE ablation in 12-month-old mice using Evans blue staining, whereas no vascular perfusion was observed in the RF-treated region [23]. Although IRE is theoretically considered to spare extracellular matrix structures, animal studies have reported minor histopathological alterations in nearby vessel walls, including vasculitis and slight endothelial injury [24,25]. In a retrospective analysis, Dollinger et al. [26] evaluated 43 patients with 84 hepatic lesions located adjacent to venous structures after IRE treatment. Vascular alterations were identified in 19 out of 191 vessels (9.9%), comprising partial portal vein thrombosis (n = 2), complete portal vein thrombosis (n = 3), and luminal narrowing (n = 14).

The safety equivalence finding is reassuring but requires contextual interpretation. Verloh et al. [19] contributed most of the weight to the complication analysis and compared IRE to a combined RFA/MWA thermal arm, potentially diluting group differences. We have included this study because the majority of the subjects received RFA, and considering the low number of studies, we included this in the meta-analysis. More clinically meaningful is the finding from the study by Zhang et al. [22] that device-specific treatment-related adverse events were observed exclusively in the IRE arm (7.9%), including subcapsular haematoma, haemocholecyst, and severe fever. Beyond formal complication rates, IRE carries additional practical burdens: it requires general anaesthesia with neuromuscular blockade (RFA is typically performed under conscious sedation), multiple parallel electrode placement demanding greater procedural precision, and significantly longer procedure time (34.3 versus 19.9 min; p<0.001 in [22]). These differences have implications for resource allocation, scheduling, and patient selection.

The substantial heterogeneity observed in the LTRP analysis (I2=64%) warrants careful consideration. Several sources likely contribute. First, the definition of LTRP was not uniform across studies: some studies defined it as radiological evidence of residual or recurrent enhancement at the ablation site, while others used broader locoregional recurrence definitions incorporating satellite nodules. Second, the follow-up periods varied considerably, from six months in Zhang (2022) [22] to 24 months in Liu (2022) [18], making direct comparison of event rates inherently imprecise. Third, patient populations differed substantially: Wang et al. [21] enrolled exclusively subcapsular HCC, Wada et al. [20] enrolled perivascular BCLC 0/A HCC, and Zhang et al. [22] enrolled mixed HCC and metastatic disease without mandatory proximity to vascular structures. The clinical expectation of IRE benefit is substantially stronger in vascular-adjacent disease, explaining why studies focusing on these locations showed more pronounced treatment differences. This clinical heterogeneity likely underpins the statistical heterogeneity observed and is an important reason why the current pooled estimate should be interpreted as an average treatment effect across a heterogeneous mix of clinical scenarios rather than a single coherent trial-type estimate.

From a clinical practice standpoint, the current evidence supports IRE as a safe and technically effective ablative modality for hepatic malignancies, with a comparable overall complication profile to RFA [26]. The consistent directional trend towards improved local tumour control, even if not reaching conventional statistical significance, suggests that IRE may be the preferred ablative option for patients with tumours in anatomically challenging locations adjacent to major portal or hepatic venous structures, the hepatic hilum, or the liver capsule, where the heat-sink effect or risk of thermal injury to adjacent structures would compromise RFA. This aligns with current EASL guidelines, which identify proximity to critical structures as a relative contraindication to thermal ablation [27]. In patients with technically feasible tumours for both modalities, the choice between IRE and RFA should also incorporate considerations of procedural complexity, anaesthetic risk, institutional experience with IRE, cost, and the need for general anaesthesia.

A fundamental limitation of the observational studies is confounding by indication. In clinical practice, IRE is preferentially selected for patients whose tumours are not amenable to thermal ablation, meaning that IRE and RFA groups in non-randomised studies represent clinically non-interchangeable populations. IPTW (Wang 2025) [21] and PSM (Wada 2023) [20] substantially mitigate but cannot fully eliminate this bias. Crucially, this confounding operates in a direction that should bias against IRE (IRE patients have harder-to-treat tumours), making the observed LTP benefit more rather than less noteworthy. The two RCTs [18,22] provide allocation-independent evidence but studied broadly operable tumours without mandatory proximity to critical structures, precisely the cases where IRE's biological advantage is least expected to manifest.

Compared with prior systematic reviews on this topic, the present study differs fundamentally in study design eligibility. Gupta et al. [11] pooled 23 single-arm IRE studies (n=702) and reported a technical success rate of 95.0% and a major complication rate of 4.8% but could not draw any conclusions regarding comparative efficacy versus thermal ablation. Tian et al. [12] similarly pooled single-arm data from 12 studies, reporting an overall local recurrence rate of 9.8% but again without a comparator arm. Yu and Li [13] restricted their analysis to HCC specifically and included 15 single-arm studies, reporting a one-year LTP rate of 10.1% with IRE. None of these prior reviews could address the comparative effectiveness question by design, as single-arm pooling cannot control for differences in patient population, tumour selection criteria, or institutional expertise between IRE and RFA cohorts. The current analysis addresses this fundamental gap by restricting inclusion to studies where both modalities were directly compared in the same patient population, providing the only valid design for comparative inference. The non-significant trend in LTRP (RR 0.68) identified here is directionally consistent with the hypothesis that IRE may offer superior local tumour control but requires confirmation in larger prospective comparative studies.

This meta-analysis has several important limitations. The primary limitation is the small number of included studies per outcome (k=2-4), resulting in limited statistical power and wide confidence intervals that preclude definitive comparative conclusions. The total sample size for LTRP analysis was modest (n=405 across four studies). The eligible evidence base comprises two RCTs and four retrospective cohort studies, with the latter subject to confounding by indication, a bias that operates in the direction of making IRE appear worse (since IRE is selected for harder-to-treat tumours), and thus is unlikely to fully explain any observed benefit. Propensity score methods used in Wang et al. [21] and Wada et al. [20] substantially mitigate but cannot eliminate residual confounding. Heterogeneity in outcome definitions, imaging protocols, follow-up duration, and tumour selection criteria further limits the interpretability of pooled estimates. The study by Verloh et al. [19] included a mixed thermal comparator (RFA/MWA), and the RCT by Liu et al. [18] enrolled only 16 patients per arm, contributing imprecise estimates. Finally, given that fewer than five studies contributed to any outcome, formal assessment of publication bias was not feasible [28], and the possibility of small-study effects or selective reporting cannot be excluded.

Future research should prioritise adequately powered multicentre RCTs directly comparing IRE versus RFA, with pre-specified stratification by tumour location (perivascular versus non-perivascular), standardised imaging-based LTRP definitions using modified RECIST or EASL criteria, and a minimum follow-up of two years to capture late recurrence events. Patient-level data meta-analysis, incorporating individual tumour characteristics, would allow more meaningful subgroup analyses by location, tumour type, and size than is possible with aggregate data. Health economic analyses comparing the full costs of IRE (including anaesthetic, device, and procedural time) with RFA are also warranted to inform commissioning decisions, particularly in resource-limited settings.

## Conclusions

This systematic review and meta-analysis compared the efficacy and safety of IRE versus RFA for hepatic malignancies across all available direct comparative studies. Pooled analysis demonstrated that IRE and RFA produce comparable complication rates and mortality outcomes, with no statistically significant difference between the two modalities in either domain. A consistent trend towards lower LTRP was observed with IRE compared to RFA, although this did not reach statistical significance, likely reflecting the limited number of available comparative studies and the substantial heterogeneity present across included studies in terms of patient population, tumour location, follow-up duration, and outcome definition. These findings suggest that IRE is a safe and effective ablative alternative to RFA for hepatic malignancies, with a particular potential advantage in anatomically challenging locations where thermal ablation is limited by the heat-sink effect or risk of injury to adjacent critical structures. Nonetheless, IRE is not feasible in all cases, and the procedure had to be abandoned in a small number of instances, underscoring that its advantage in such locations is not absolute. However, the current evidence base remains insufficient to draw definitive comparative conclusions, and the results of this analysis should be considered hypothesis-generating pending confirmation from larger, prospectively registered randomised trials with standardised outcome reporting and adequate long-term follow-up.
